# Examining the epigenetic transmission of risk for chronic pain associated with paternal post-traumatic stress disorder: a focus on veteran populations

**DOI:** 10.1038/s41398-025-03267-w

**Published:** 2025-02-05

**Authors:** James Freeman, Sabrina Salberg, Melanie Noel, Richelle Mychasiuk

**Affiliations:** 1 Department of Neuroscience, Monash University, Melbourne, VIC Australia; 2 Department of Psychology, University of Calgary, Calgary, AB Canada

**Keywords:** Epigenetics and behaviour, Psychiatric disorders

## Abstract

Chronic pain is a public health problem that significantly reduces quality of life. Although the aetiology is often unknown, recent evidence suggests that susceptibility can be transmitted intergenerationally, from parent to child. Post-traumatic stress disorder (PTSD) is a debilitating psychological disorder, often associated with chronic pain, that has high prevalence rates in military personnel and Veterans. Therefore, we aimed to characterise the epigenetic mechanisms by which paternal trauma, such as PTSD, is transmitted across generations to confer risk in the next generation, specifically focusing on Veterans where possible. Numerous overlapping neurological pathways are implicated in both PTSD and chronic pain; many of which are susceptible to epigenetic modification, such as DNA methylation, histone modifications, and RNA regulation. Hence, epigenetic changes related to pain perception, inflammation, and neurotransmission may influence an individual’s predisposition to chronic pain conditions. We also examine the effects of PTSD on parenting behaviours and discuss how these variations could impact the development of chronic pain in children. We highlight the need for further research regarding the interactions between paternal trauma and epigenetic processes to ultimately generate effective prevention and therapeutic strategies for Veterans who have been affected by PTSD and chronic pain.

## Introduction

Chronic pain and post-traumatic stress disorder (PTSD) are prevalent and debilitating conditions that often co-occur within both Veteran and civilian populations [1–4]. PTSD that results from traumatic experiences can have a significant influence on epigenetic processes such as DNA methylation and chromatin remodelling, often leading to persistent changes in gene regulation and gene expression [5, 6]. Epidemiological studies have found a median chronic pain prevalence of up to 38% in youth between the ages of 2 and 17 years old, with many chronic pain conditions being resistant to treatment strategies [7, 8]. Additionally, children with chronic pain symptoms are at an increased risk for persistent chronic pain that extends into adolescence and adulthood [9, 10]. Research suggests that epigenetic processes and modifications, similar to those influenced by PTSD, also play a significant role in the risk for, and development of, chronic pain conditions [11, 12]. Evidence also indicates that chronic pain and PTSD involve overlapping neurological circuitry, with aspects of pain pathways and the hypothalamic-pituitary-adrenal (HPA) axis stress response interacting synergistically to escalate both pain and PTSD symptoms [1, 3, 4, 13]. Due to the dangerous and stressful aspects of a career in the armed forces, Veterans are disproportionately exposed to traumatic situations (e.g., combat, violence, war, and exposure to death) and therefore exhibit higher prevalence rates of PTSD [1, 2, 14, 15].

This results in two key questions; how does paternal trauma, such as PTSD, influence epigenetic processes and intergenerational risk for chronic pain? Furthermore, how do variations in parenting style interact with epigenetic modifications to exacerbate or protect against chronic pain outcomes in youth? Therefore, the purpose of this review is to characterise the epigenetic mechanisms by which paternal traumas, such as PTSD, are transmitted across generations to confer risk for chronic pain in the next generation, with a specific focus on Veterans. We also examine the effects that PTSD has on parenting styles and relate these to child neurodevelopment, coping strategies, and susceptibility to chronic pain.

## Post-traumatic stress disorder (PTSD)

Although it is possible for trauma to occur and manifest in response to a variety of experiences (e.g., war/combat, vehicle accidents, abuse, neglect, hospitalisations, death), all of which have the potential to influence epigenetic processes associated with a multitude of psychological disorders (e.g., PTSD, anxiety disorders, depressive disorders), this review will focus on PTSD.

### Overview of PTSD

PTSD is a chronic and debilitating psychological disorder that stems from exposure to one or more extreme traumatic events, leading to the development of characteristic symptoms [16]. These events can include exposure to war or combat, threatened or actual physical assault, threatened or actual sexual assault, natural or man-made disasters, severe vehicular accidents, as well as witnessing serious injury or death [16]. The characteristic symptoms of PTSD can be categorised into four clusters of symptoms as outlined in the DSM-V [16, 17]. These clusters encompass: A) intrusion symptoms such as recurrent, intrusive, and distressing memories, nightmares, or flashbacks of the traumatic event; B) avoidance symptoms which involve avoiding distressing memories, thoughts, or feelings related to the traumatic event, or avoidance of external reminders that trigger distressing memories, thoughts or feelings of the traumatic event; C) emotional numbing symptoms, which manifest as dissociative amnesia, negative self-beliefs, negative emotional states, feelings of detachment, and anhedonia; and D) hyperarousal symptoms, including irritable or angry behaviour, self-destructive tendencies, hypervigilance, exaggerated startle response, and sleep disturbances [16]. PTSD is commonly associated with increased risk of suicidal ideation and attempts, and often coexists with other psychiatric comorbidities such as major depressive disorder, anxiety disorders, insomnia, and alcohol or substance abuse [18, 19]. Though the symptomatology of PTSD is broadly categorised into four clusters (intrusion, avoidance, emotional numbing, and hyperarousal symptoms), the frequency, nature, and intensity of these symptoms can significantly vary among those affected. PTSD symptom diversity is complicated further by its temporal manifestation. The heterogeneous nature of PTSD symptom manifestation is associated with a multitude of factors, such as the severity of the trauma, previous psychological history, genetic predisposition of the individual, as well as their coping mechanisms. Furthermore, differences in neurobiological responses to trauma can also contribute to the diverse range of symptom manifestations [20, 21]. This variability highlights the intricate nature of PTSD and challenges traditional standardised therapeutic approaches to diagnosis and treatment.

### Prevalence of PTSD

Although prevalent across cultures and contexts, owing to their significant exposure to trauma during training exercises and combat in war zones, military personnel and Veterans are at particular risk of developing PTSD [2, 22]. A review that investigated PTSD prevalence among both civilian and Veteran populations found that PTSD prevalence among civilians was 11.1%, whereas PTSD prevalence among Veterans was 24.5% [23]. Among current literature reporting PTSD prevalence among U.S. help-seeking Veterans, the prevalence of PTSD ranged from 9.5–41.3%. However, a meta-analysis conducted by Fulton et al. found that the average prevalence rate of PTSD among U.S. Veterans who served in Iraq or Afghanistan was 23% [24], and soldiers who were deployed on operations twice were 60% more likely to develop PTSD compared to soldiers with one deployment [25]. Similar prevalence rates of PTSD have been reported among Australian Defence Force soldiers, between 8 and 21.8% [26, 27]. Additionally, in a sample of 200 soldiers who suffered a traumatic brain injury, PTSD prevalence rates were as high as 68% [28].

### Neurobiology of PTSD

**Fig. 1:**
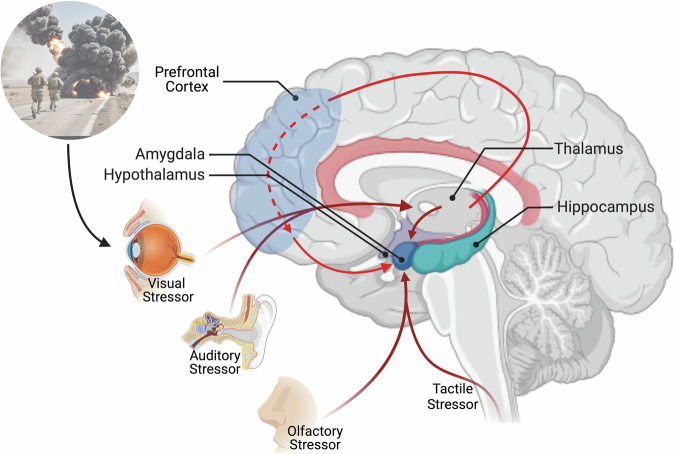
Cortical pathways involved in processing traumatic experiences. Exposure to trauma and stressors are first processed by the thalamus and relayed to the PFC for regulation of the fear response. The stimuli are then transmitted to the amygdala to respond to the danger (stress response/fight or flight). The amygdala assesses incoming information for potential threats and triggers the appropriate emotional and behavioural response for survival when under threat. Figure was created with BioRender.com.

The neurocircuitry and neurophysiology responsible for both the establishment and maintenance of PTSD are extensive. Therefore, we have focused on some of the primary constituents and their associated functions. See Fig. 1 for a diagrammatic illustration. The amygdala plays a pivotal role in processing and regulating emotional responses, whereby particularly in the presence of a threat, it can activate the body’s fight-or-flight/stress response [29].

**Fig. 2:**
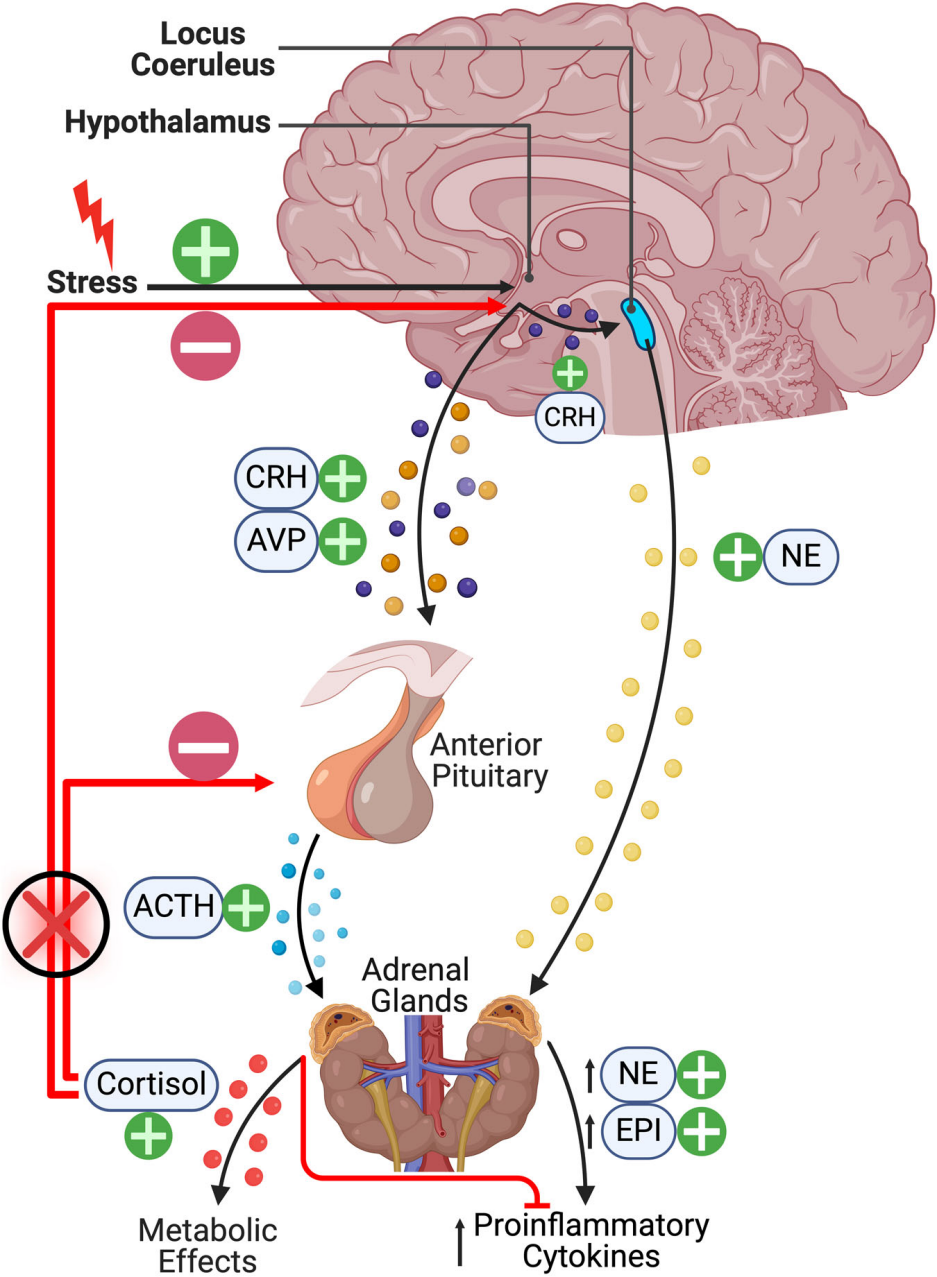
Stress-induced activation of the HPA axis stress response. The hypothalamus secretes AVP and CRH, which travel via the hypophyseal portal system to stimulate secretion of ACTH from the anterior pituitary. ACTH enters circulation and stimulates the release of cortisol from the adrenal medulla, resulting in metabolic effects such as gluconeogenesis. Cortisol inhibits the hypothalamus and anterior pituitary from secreting CRH and ACTH, respectively, working as a negative feedback mechanism. In PTSD, this negative feedback mechanism is impaired, resulting in increased CRH and ACTH as well as inflammation. Figure was created with BioRender.com.

Under typical conditions, exposure to an extreme stressor results in the activation of the autonomic nervous system (ANS) and the HPA axis [30]. As demonstrated in Fig. 2, within the context of PTSD, glucocorticoid signalling is dysregulated and the HPA axis negative feedback loop is inhibited, resulting in increased corticotropin-releasing hormone (CRH) release but blunted adrenocorticotropic hormone (ACTH), resulting in diminished secretion of cortisol [31]. The hypocortisolemia identified in PTSD may be the result of over-suppression of the HPA axis negative feedback. In a study of Vietnam combat Veterans, metyrapone was administered, and ACTH secretion from the pituitary was examined. Metyrapone administration caused a significant increase in ACTH and 11-deoxycortisol when compared to the control group, providing support for the negative feedback inhibition hypothesis [32].

### Allostatic load

Allostasis refers to the body’s physiological mechanisms that are responsible for managing homeostasis during demanding or stressful events, while allostatic load refers to the cumulative burden of chronic stressors, that often do not terminate [33]. In response to being exposed to stressful, threatening, or traumatic situations, allostatic mechanisms are adaptive and attempt to re-establish balance [34]. When the brain deems a situation to be a threat, the brain triggers physiological and behavioural responses via neurological pathways involving the thalamus, PFC, amygdala, and hypothalamus, as well as endocrine pathways such as the HPA axis [34–36].

**Fig. 3:**
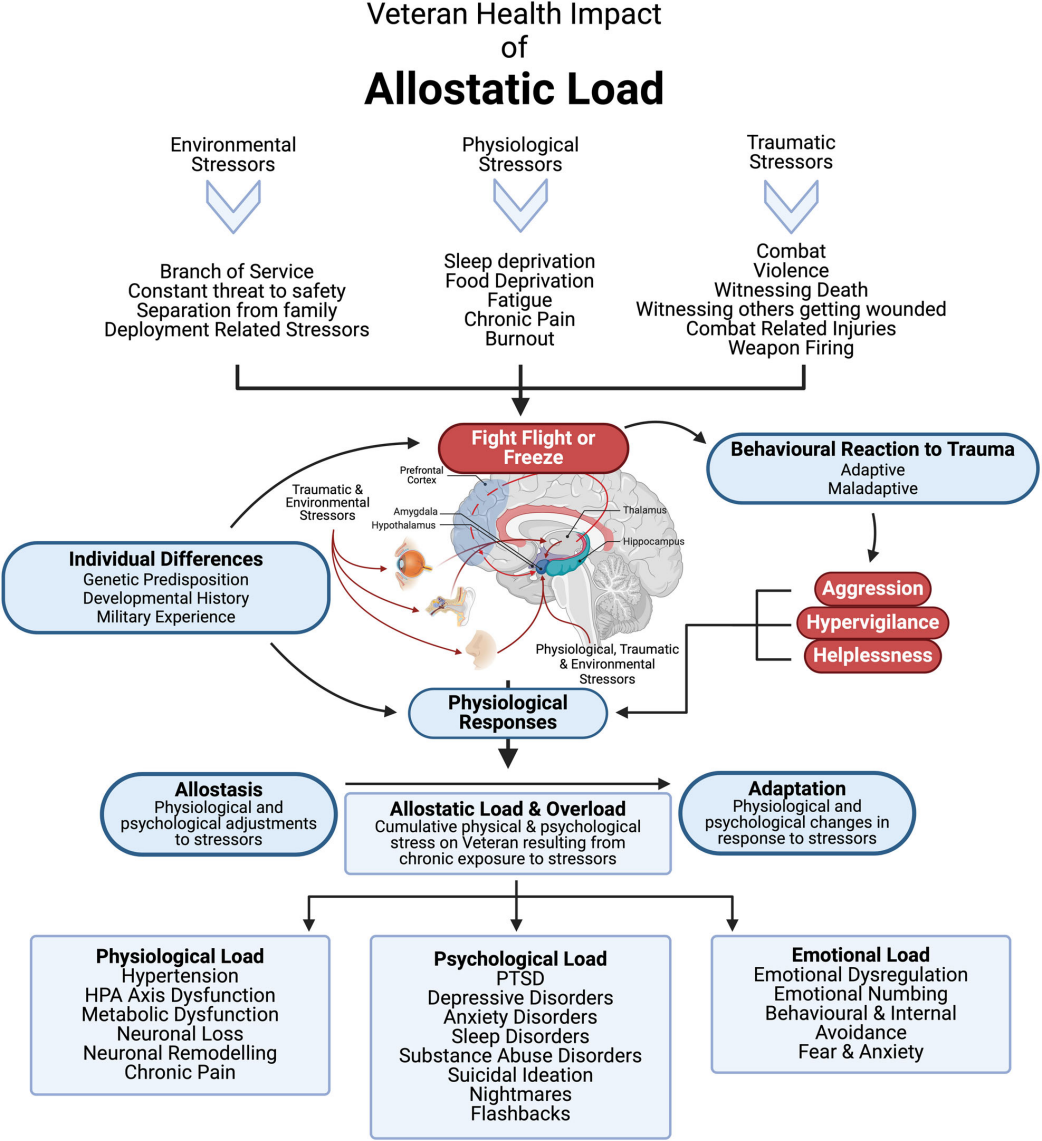
Allostatic load is affected by numerous physiological, genetic, and environmental factors that influence the behavioural response to stress and fear. These factors consequently influence neurotransmitter and hormone concentrations in the brain, resulting in a physiological response that contributes to physical and psychological changes that result in clinical pathologies such as PTSD, sleep disorders, and metabolic dysfunction. Figure was created with BioRender.com.

For Veterans, response to stressors, like other individuals, are dependent on factors such as developmental history (e.g., early life stress), current environmental situations, and genetic predisposition [37]. However, military training and combat experience also play a significant role in influencing their behavioural responses to stressors [38]. Within military personnel and Veteran populations, allostatic load accumulates as a result of the increasing physiological and psychological degradation due to the body’s continuous adjustment to ongoing stressors [39, 40]. Contributing factors to allostatic load can be physiological, such as HPA axis dysfunction [41] decreased hippocampal volume, and remodelling of the PFC and amygdala [34]. Psychological load is another factor and can include hyperarousal, hypervigilance, impaired cognitive functioning, and sleep disturbances [39]. Lastly, emotional load can involve emotional dysregulation and impaired fear extinction [33]. These contributing factors can lead to allostatic overload, whereby the individual experiences adverse effects such has cardiovascular disease, due to the accumulation of stressors [41] See Fig. 3.

## Chronic pain

### Prevalence and overview of chronic pain

Chronic pain is a debilitating condition that affects 11–38% of children between the ages of 2–17 years [42] and 11–31% of adults [43], making chronic pain a global health issue that significantly contributes to decreased quality of life [7]. A meta-analysis derived from 23 chronic pain studies that included global populations of adults between 18–75 years of age, calculated a pooled chronic pain prevalence of 11.8% [44]. Although chronic pain rates are generally relatively high, they vary with age. The Australian Institute of Health and Welfare (AIHW) found that 19% of Australians over 45 experience chronic pain, with chronic pain increasing to 24% in people over the age of 85. AIHW also found that women suffered higher rates of chronic pain (21%) compared to men (17%) [45]. Of importance, chronic pain is often a sequela of PTSD, with prevalence rates of PTSD among individuals of the general population suffering from chronic pain being 9.7% [15] and 9.8% [3]. Alarmingly, among the Veteran population, the prevalence of chronic pain in Veterans suffering from PTSD was 66% [46].

### Neurobiology of chronic pain

**Fig. 4:**
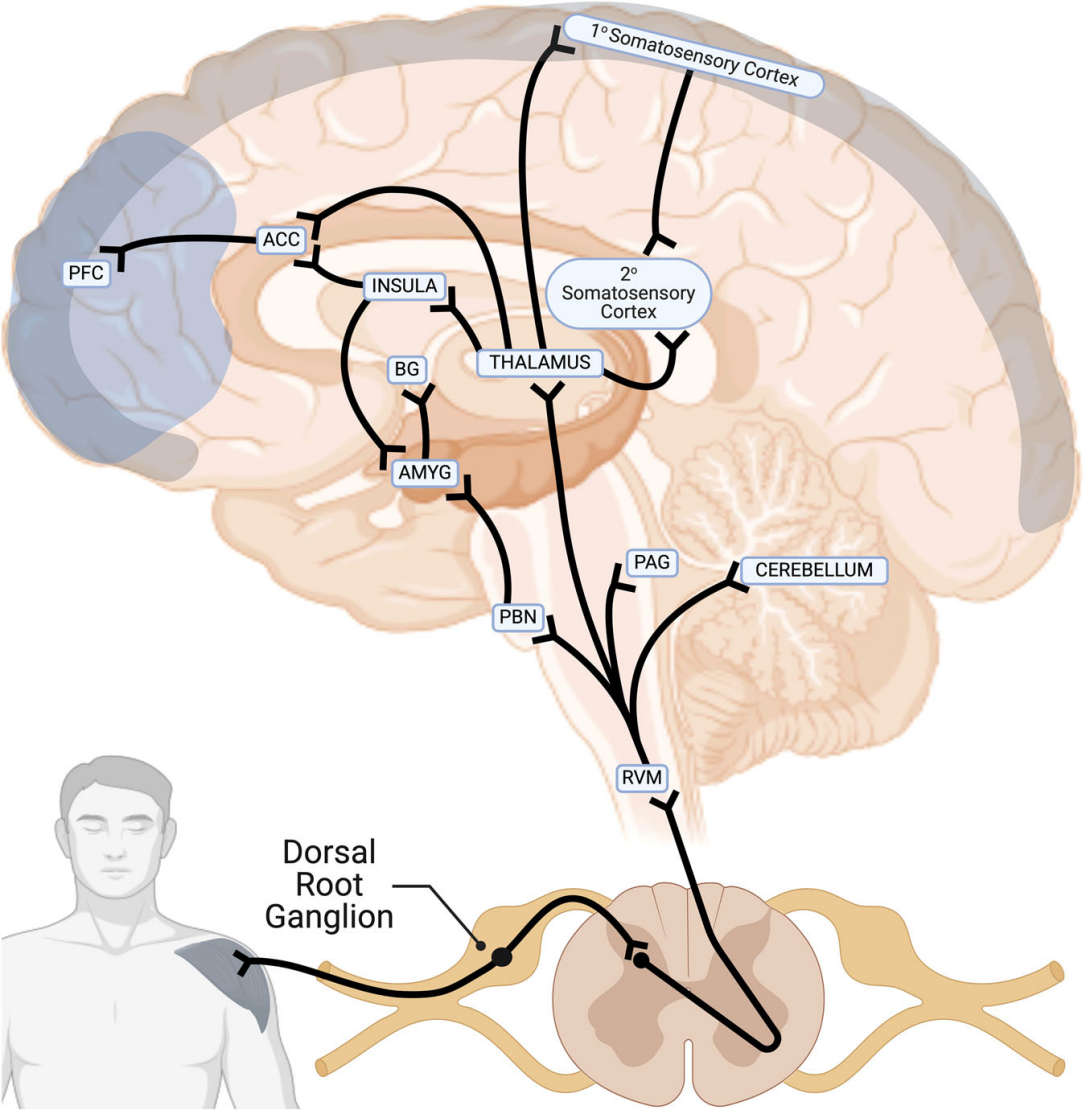
Primary afferent nociceptors transmit noxious stimuli via the dorsal root ganglion to projection neurons situated in the spinal cord’s dorsal horn. A subgroup of these projection neurons transmits noxious stimuli to the thalamus which is then relayed to the somatosensory cortex. The somatosensory cortex is responsible for the location and quality of the noxious stimuli. The parabrachial nucleus and the amygdala transmit noxious stimuli to the ACC, basal ganglia (BG), insula, and PFC, which play a significant role in regulating the emotional and affective aspects of the pain experience. Figure was created with BioRender.com.

Pain is classified as either nociceptive, neuropathic, or inflammatory. However, all types of pain can coexist [47, 48]. To fully appreciate the relationship between PTSD and chronic pain, we need to delve into the neurobiological processes driving the manifestation of persistent pain states. When an ample amount of primary afferent nociceptors are activated succeeding a painful stimulus, an action potential is generated, and the afferent noxious pain stimuli are transmitted from peripheral primary nociceptors to the dorsal horn of the spinal cord [49]. The emotional affective aspects of pain emanate from the superficial dorsal horn and travel upward through the parabrachial region to synapse with various brain structures responsible for discomfort, such as the PFC, periaqueductal grey (PAG), and the anterior cingulate cortex (ACC) [50]. These physical and emotional properties of pain activate downstream pathways that play a role in the epigenetic regulation and transcription of genes involved in facilitating and subsequently maintaining pain within chronic pain states, such as central sensitization. See Fig. 4 for an illustrative representation of this connectivity.

Central sensitisation is a neurological condition that occurs in the central nervous system (CNS) resulting in hyperalgesia (caused by increased sensitivity to a painful stimulus) and allodynia (pain caused by normally non-painful stimuli) [51]. Physiologically, central sensitisation is the result of reduced threshold, increased responsiveness and excitability, and expanded receptive fields of neurons in the dorsal horn. Consequently, low-threshold neurons activated by innocuous stimuli are now able to act on high-threshold nociceptive neurons [52, 53].

**Fig. 5:**
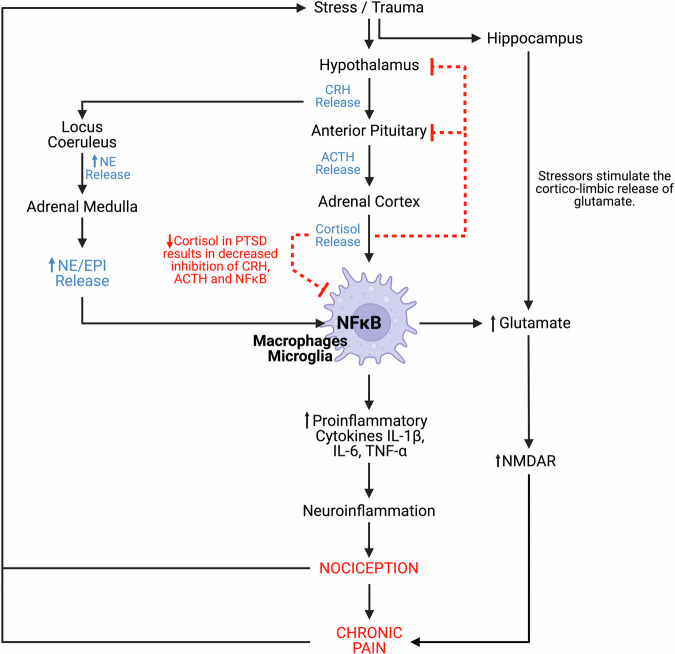
Due to the dysregulation of the HPA axis there is a decrease in cortisol concentrations and an increase epinephrine (EPI) and NE production, the inhibition of immune cells is discontinued, and production of proinflammatory cytokines and glutamate is upregulated. Consequently, this results in the manifestation of chronic pain states, and ultimately, chronic pain itself becomes a stressor, resulting in a perpetuated and dysregulated HPA axis response. Figure was created with BioRender.com.

Prolonged inflammatory responses triggered by repetitive nociceptive stimulation in peripheral tissues have significant implications for pain perception and central sensitisation [54]. Allodynia, referring to pain in response to a stimulus that does not normally provoke pain, is a common symptom of chronic pain, often resulting from changes to peripheral sensitisation, as demonstrated in Fig. 5 [51]. Repetitive nociceptive stimulation in peripheral tissues can lead to a prolonged inflammatory response by activating lymphocytes and triggering the release of proinflammatory molecules [55]. This chronic inflammation results in a reduced pain threshold, upregulation of Na+ channels, and increased substance P production [56]. The spinal cord also releases neurotransmitters such as glutamate, ATP, and substance P, as well as neuromodulators such as nitric oxide (NO) and prostaglandins that ultimately activate glial cells in the CNS [56, 57]. Glial cells release proinflammatory molecules interleukin (IL) IL-1, IL-6, and tumor necrosis factor alpha (TNF-α) in addition to (NO), chemokines, prostaglandins, and reactive oxygen species (ROS) in the CNS. The release of these substances causes an up-regulation of NMDA receptors and down-regulation of GABA receptors, increasing neuronal excitability and ultimately resulting in central sensitisation [56, 57]. In summary, the transition from acute to chronic pain is a complex process involving neurobiological and physiological changes. In cases where continuous and repetitive nociceptive stimulation results in pathophysiological changes in pain processing pathways, acute pain can transition into chronic pain [47, 54].

## Epigenetic mechanisms in gene expression

There is a growing need to understand how experiences such as PTSD in Veterans are transmitted across generations and get under the skin of offspring to produce long-lasting change. Epigenetics have traditionally been described as the dynamic interplay between the genome and the environment that result in variations to gene expression and phenotype [58]. Importantly, many traumatic experiences have the ability to modulate epigenetic processes, thereby changing offspring gene expression and neurodevelopment. Epigenetic regulation of gene expression can be modulated during gene transcription or translation via multiple mechanisms, including DNA methylation, histone modifications, and RNA regulation involving RNA interference (RNAi). These epigenetic modifications control the state of chromatin compaction and access to transcription factors, thereby promoting cellular differentiation and specialisation [59]. In a similar fashion to other environmental factors, both maternal and paternal social experiences can influence the development of their offspring. A plethora of research has demonstrated that prenatal maternal stress, nutrition, and drug use can result in physiological changes in offspring. For review, see Champagne, 2010. Furthermore, the development of the HPA axis response to stress is often negatively impacted when offspring are exposed to postnatal maternal separation or withdrawal of maternal care [60]. Evidence indicates that these changes in the stress response result from epigenetic programming [61].

Although less well studied, paternally-generated programming processes, such as exposure to stress, can also result in epigenetic variation within developing spermatozoa and have the potential to be epigenetically transmitted to their offspring [62]. Within species that co-parent, paternal absence during offspring development can also cause HPA axis dysregulation and increased sensitivity to stressors [63].

### DNA methylation

DNA methylation is the primary epigenetic mechanism utilised during development and is indirectly responsible for the suppression of transcriptional activity [64]. DNA methylation involves multiple layers, the first is an enzymatic reaction catalysed by DNA methyltransferase (DNMTs) enzymes that convert cytosine nucleotides to 5-methylcytosine [65, 66]. Although beyond the scope of this review, there are multiple means by which DNA methylation represses transcription, thereby preventing the expression of specific genes.

### Histone modification

A second mechanism employed to regulate gene expression is histone modification. Condensed chromatin formation (heterochromatin) inhibits DNA access, preventing transcription [67–69]. Histone acetylation is involved in transcription activation and is mediated by histone acetyltransferase (HAT) enzymes. HATs catalyse the transfer of acetyl groups from acetyl-CoA to lysine amino acids on histone N-terminal tails, negating the positive charge of lysine and weakening the bonds between DNA and histones [70]. Conversely, histone deacetylase (HDACs) enzymes reverse HAT-mediated histone/lysine acetylation, promoting transcriptional repression by changing chromatin back to the heterochromatic form [70, 71]. HDACs also associate with the transcriptional repressor domain of MeCP2, synergising two gene suppression mechanisms; DNA methylation and histone deacetylation [72].

Histone phosphorylation is associated with the regulation of gene expression via phosphorylation of histone H3, and chromatin compaction via phosphorylation of serine on histone H3 N-terminal tails [73]. During mitosis and meiosis, histone N-terminal tail serine and threonine phosphorylation is associated with chromatin condensation and, therefore, transcription inhibition [74]. Histone ubiquitination involves the attachment of ubiquitin to a lysine residue of the N-terminal tails of histones, either via a direct or indirect pathway [75, 76]. Chromatin containing ubiquitinated H2B (H2B-Ub) promotes an open chromatin formation, allowing DNA replication and gene transcription to occur [77]. Sumoylation is a pivotal post-translational modification that is catalysed by the Small Ubiquitin-like Modifier (SUMO), a protein modifier belonging to the ubiquitin-like protein (Ubl) family [78] that regulates gene transcription, DNA replication, cell cycle regulation, and DNA repair. SUMO operates in a similar function to and is structurally related to ubiquitin, whereby increased sumoylation results in gene expression [78, 79].

### RNA regulation

RNA regulation is mediated by small noncoding RNA (ncRNA) and long-noncoding RNA (lncRNA) that regulate gene expression. Some types of small ncRNA include short interfering RNA (siRNA), microRNA (miRNA), and PIWI-interacting RNA (piRNA) [64]. MicroRNAs are single-stranded RNAs derived from intergenic and intronic regions of protein-coding genes. The function of miRNA is to integrate into and activate the miRNA-induced silencing complex (miRISC) [80]. The miRNA repress post-transcriptional gene expression by imperfect base-pairing with target complementary messenger RNA (mRNA) causing inhibition of translation or, by perfect base-pairing that promotes Ago2 endonuclease cleavage resulting in mRNA degradation and post-transcriptional gene silencing [80, 81]. siRNAs are double-stranded RNA (dsRNA) molecules that regulate gene expression and are involved in RNA interference (RNAi), which is responsible for post-transcriptional silencing of gene expression. Within this pathway, siRNA interfaces with the RNA-induced silencing complex (RISC), catalysing the cleavage and degradation of mRNA and miRNA, resulting in the inhibition of translation [81].

## Investigating paternal epigenetic transmission

Studies of paternal effects indicate that gametic epigenome changes that arise because of environmental factors and life experiences can impact offspring development and disease susceptibility [58, 62, 63, 82–85]. Intergenerational paternal transmission of chronic pain is a complex phenomenon in which susceptibility to chronic pain conditions is inherited from fathers to their offspring. While maternal contributions to offspring phenotype have been extensively studied, this phenomenon has sparked interest and research into the paternal contributions of pain epigenetics, which may permit the future elucidation of potential mechanisms driving the manifestation of chronic pain across generations. It has been well established that offspring of parents suffering from chronic pain are at increased risk of developing not only chronic pain but also psychological comorbidities such as depression, anxiety, and PTSD [86, 87].

One mechanism through which paternal environmental exposures can influence offspring neurodevelopment is via epigenetic marking of germ cells [88]. The paternally imprinted epigenetic modifications, also known as genomic imprinting, are epigenetic control mechanisms that exhibit specific parent-of-origin gene expression patterns by methylating CpG-rich domains in parental genes [63, 70, 74, 89]. Genomic imprinting leads to monoallelic gene expression by selective expression of one of the two parental alleles [90], with paternally expressed genes being inherited in a patrilineal fashion and maternally expressed genes being inherited in a matrilineal fashion [91]. There are currently 129 known human imprinted genes with a large proportion of these being expressed in the CNS and playing significant roles in neurodevelopment, in both the prenatal and post-natal periods, as well as in behaviour and brain function [90, 91].

During gonadal sex determination, the germ line cells undergo complete demethylation, which deletes previous parental-specific methylation of genes, involved in the regulation of imprinted gene expression. During early embryogenesis, there is active demethylation of the paternal genome as well as simultaneous gradual passive demethylation of the maternal genome, both of which occur in response to decreased *DNMT1* activity [92–94]. Following embryogenesis, both the paternal and maternal genomes undergo genome-wide re-methylation at implantation [92, 95]. Interestingly, however, throughout sex determination and embryogenesis, imprinted genes retain their methylation marks, and thus, the inheritance of parental-specific phenotypic expression occurs [92–94].

**Fig. 6:**
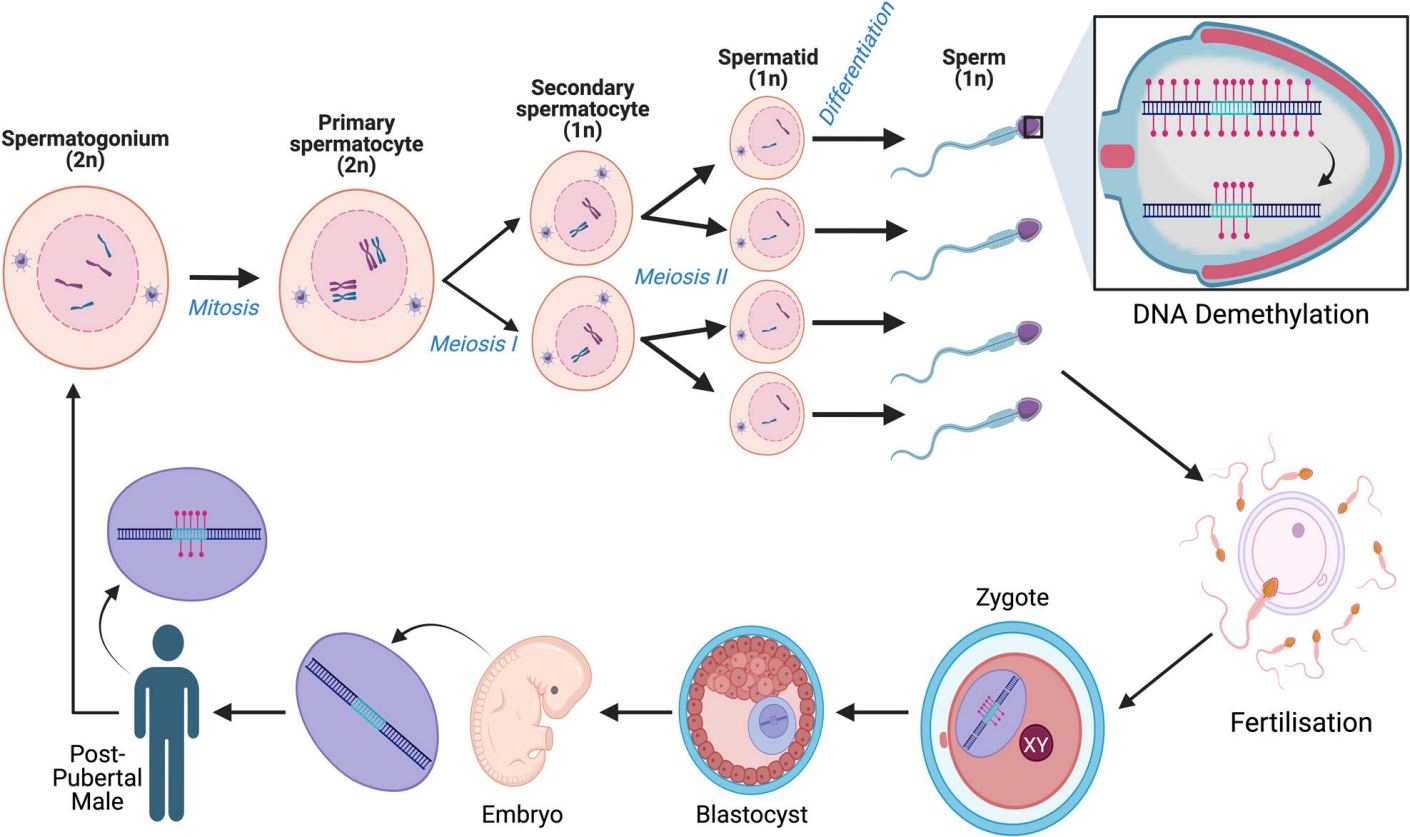
The global DNA methylation process throughout the paternal life cycle. The human spermatogenesis process begins at puberty, with each cycle lasting approximately 64 days. Following fertilisation genome-wide DNA demethylation occurs within the zygote, with the exception of epigenetically inherited imprints. Progressive demethylation continues until the blastocyst stage, when DNA methylation levels are at their lowest just prior to implantation. DNA methylation patterns are then re-established following implantation. A second round of DNA demethylation that includes the demethylation of epigenetically inherited imprints occurs in primordial germ cells at the end of trimester 1, where they now have the least methylated genome of the entire lifespan. However, epigenetically inherited imprints are protected by an unknown mechanism from this second round of genome-wide demethylation [155, 156]. Following the second round of DNA demethylation, DNA re-methylation occurs, which continues into the post-natal development stages. Figure was created with BioRender.com.

Paternal experiences that have been demonstrated to induce epigenetic changes include trauma, pain, stress, toxins, alcohol use, drug use, nutrition, and exercise [63]. Past studies that have utilised rodent models for paternal exposure to stress paradigms provide evidence that paternal experiences can epigenetically modify genes in offspring via epigenetic marks on spermatozoa during spermatogenesis [84, 96]. Unlike oogenesis, spermatogenesis begins at puberty and is a continuous process throughout the male lifespan. Therefore, spermatogenesis may actually render paternal lineages more susceptible to epigenetic programming via environmental factors and experiences, thereby having greater influences on offspring development [83]. See Fig. 6. More specifically, paternal experiences have been found to result in the intergenerational transmission of stress-induced depressive and anxiety-like behaviours, increased cortisol levels, HPA axis dysregulation, and chronic pain [96]. For example, using a mouse model of chronic stress, researchers discovered numerous changes to epigenetic marks in germline cells. These changes included modifications to nine sperm miRNAs that resulted in epigenetic reprogramming of the HPA axis stress response in the offspring [61]. An additional rodent study demonstrated that the progeny of chronically stressed sires displayed an increase in both depression and anxiety-like behaviours, and the male offspring also had increased baseline corticosterone levels, as well as decreased vascular endothelial growth factor (*VEGF*) expression [96]. Furthermore, in human sperm samples, principal component 2 analyses of miRNA was able to differentiate samples from different stress phenotypes, and in vitro, immortalized mouse distal caput epididymal epithelial extracellular vesicles (EVs) exhibited changes to miRNA expression pattern, EV protein content, and vesicle size following stress treatment [97]. Therefore, paternal experiences such as stress prior to conception can result in significant consequences for offspring development and potentially lead to adverse outcomes later in life.

### Gene-trauma-epigenetic interactions in PTSD and chronic pain development

As mentioned above, it is well established that paternal physical exposures can be inherited, however this section will focus on the evidence associated with paternal trauma/PTSD and/or offspring outcomes related to chronic pain. With respect to genetic factors, there is considerable evidence from longitudinal cohorts and genome-wide association studies (GWAS) that genes play pivotal roles in both the manifestation and intergenerational transmission of risk for PTSD and chronic pain [6, 11, 30, 98–102]. In addition, evidence suggests that some individuals carry genetic predispositions that contribute to increased susceptibility to PTSD and chronic pain following exposure to a traumatic experience [61, 103, 104]. Paternal transmission of psychological experience to offspring is less well characterized, however research in this area is expanding. In an excellent review, Raza et al., compiled evidence demonstrating that stress, depression, emotional/sexual trauma, and lifetime PSTD were associated with modifications to DNA methylation of *NR3C1*, also known as *GR*, while early life trauma and PTSD also altered methylation of *FKBP5*, a regulator of intracellular GR signalling [105]. Moreover, DNA methylation changes in numerous genes (*BDNF, MAOA, LHX1, PAX8, AHRR, ZKP57, RNF39, HIST1H2APS2*) have been identified in known populations of individuals with PTSD [106–109]. For example, survivors of the 9/11 terrorist attacks in the USA, who have been diagnosed with PTSD, had differential expression in 25 genes associated with HPA axis, immune, and cerebral function [110].

Additionally, heritable epigenetic variations in specific genes related to pain perception and neurotransmission may also influence on an individual’s predisposition to developing chronic pain conditions [48]. For example, SNPs in the *FKBP5* gene that encodes the co-chaperone heat shock protein 90 (HSP90), which regulates cortisol binding to glucocorticoid receptors, have been linked to a heightened risk for developing psychopathologies such as PTSD, as they alter glucocorticoid sensitivity and stress hormone regulation [111]. Furthermore, childhood trauma can result in decreased methylation of the *FKBP5* promoter region which is then associated with an increased risk of developing PTSD in adulthood. In individuals who experience increased chronic stress, SNPs present in *FKBP5* increase musculoskeletal pain severity following a traumatic event [112]. Directly relating this to PTSD, a genome-wide scan of sperm epigenomes in Vietnam War Veterans, provided insight into the heritability of PTSD via epigenetic changes [113]. The researchers linked 33,129 CpG sites to PTSD diagnosis, including DNA methylation at two sites in *NR3C1* and two additional sites in *FKBP5*. Interestingly, DNA methylation at 16 CpG sites in *NR3C1* and four sites in *FKBP5* in paternal sperm were associated with a reported mental health diagnosis in offspring. Of note, 62 genes with this association to reported mental health diagnosis were genes resistant to demethylation in sperm, thus making them promising candidates for transgenerational inheritance [113]. Trauma has also been found to induce histone modification changes that can influence gene expression [68] of proinflammatory genes and genes related to PTSD and chronic pain [114]. IL-1β and TNF-α levels in PTSD patients are significantly elevated when compared to healthy patients without PTSD, suggesting that individuals with PTSD are constantly in a proinflammatory state [115]. Furthermore, this proinflammatory state exacerbates chronic pain conditions as IL-1β can directly cause the excitation of nociceptive fibres and act on TRPV1, GABA, and NMDA receptors, as well as increase heat-evoked expression of CGRP [116]. At the same time, TNF-α contributes to pain by increasing mechanical hyperalgesia by sensitisation of nociceptive nerve terminals [55].

Another essential component of the epigenetic regulatory system implicated in these disorders are ncRNAs, particularly miRNAs. For example, miR-19b has been linked to both widespread pain and post-traumatic stress symptoms (PTSS) following a traumatic experience and it is possible that miR-19b predicts vulnerability to and influences widespread pain and PTSS [117]. However, the mechanistic relationship between miR-19b and PTSS and pain is currently poorly understood. Similarly, miR-132 has been linked to the process of fear extinction that is associated with PTSD. A mouse study utilising predator scent stress fear conditioning demonstrated increased miR-132 expression in the hippocampus and decreased acetylcholinesterase expression. This resulted in a prolonged anxiety and stress phenotype and cognitive impairments such as impaired learning and memory [118]. Additionally, studies have demonstrated dysregulated and increased miR-132 expression in the DRG of patients with chronic neuropathic pain, providing evidence that upregulated miR-132 expression is associated with neuropathic pain [119]. Taken together, this research indicates that miRNAs are involved in chronic pain states and neuroplasticity and, therefore, can contribute to the development of chronic pain.

Building on this, a host of research done by Yehuda and colleagues, provides strong evidence that PTSD-induced epigenetic changes can be inherited intergenerationally and may contribute to the manifestation of pathologies in the next generation [120–123]. Combat Veterans with PTSD have lower methylation levels in the promoter region of *NR3C1-1F* which is associated with lower urinary cortisol excretion, greater glucocorticoid sensitivity in peripheral blood mononuclear cells, and a greater decline in cortisol response following dexamethasone administration (DEX) [123]. This suggests dysregulation of the negative feedback inhibition loop within the HPA axis of combat Veterans with PTSD [123]. Furthermore, in veterans with PTSD that received psychotherapy, methylation of *NR3C1-1F* predicted treatment outcomes and was correlated with urinary cortisol levels and to PTSD symptom severity [121]. Gene expression of both *NR3C1-1F* and *FKBP51* were higher in those that responded to the therapy, with expression correlating to plasma cortisol levels [121]. A similar effect was identified in the offspring of Holocaust survivors who were diagnosed with PTSD, whereby they exhibited lower cortisol levels, enhanced cortisol suppression, and increased prevalence of PTSD [120]. Of note, these changes often did not depend on the trauma exposure and were instead directly correlated with parental PTSD, whereby parental PTSD, but not Holocaust exposure, was associated with the HPA axis alterations [120]. Similarly, chronic social defeat stress in adult male mice resulted in offspring depression, anxiety, and altered corticosterone and vascular endothelial growth factor levels, an effect that was not dependent on the sires response to the stressor [124].

What is particularly interesting about this research, is that the offspring effects are often dependent upon the parental exposure (maternal vs. paternal) and the offspring sex (sons vs. daughters). For example, when both parents experienced PTSD following Holocaust exposure, offspring exhibited lower DNA methylation of *NR3C1-1F* in peripheral blood cells, which was associated with enhanced cortisol suppression following DEX [122]. However, when only the father experienced PTSD, offspring exhibiter higher methylation of *NR3C1-1F*, and greater cortisol excretion and reduced GR sensitivity [122]. Moreover, paternal PTSD resulted in offspring with less adaptive attachment styles and higher rates of childhood trauma and sensitivity to violence, while maternal PTSD resulted in offspring with increased rates of depression and anxiety [122]. There were also direct sex effects in the epigenetics of those experiencing the traumatic events. In survivors of the Rwandan genocide, women exhibit greater DNA methylation of *NR3C1* than men, with methylation levels being negatively correlated with intrusive memory symptom severity and lifetime PTSD risk in men, but not women [125]. Given that activation of glucocorticoids are important for memory formation, the changes in methylation of *NR3C1* may be related to reduced memory function; as for healthy male subjects, methylation levels of *NR3C1-1F* negatively correlate with recognition memory and *NR3C1* salivary gene expression [125]. As the majority of past research regarding intergenerational transmission of risk has focused on mothers, these findings emphasize that maternal and paternal experiences are not the same, but both contribute in meaningful ways, potentially influencing offspring differently, dependent upon their sex.

The interplay between genes and traumatic experiences in the context of both PTSD and chronic pain highlights the complex nature of these conditions, opening novel avenues for future research and potential therapeutic interventions. Gaining further insight into the neurophysiological mechanisms underpinning these disorders could pave the way for efficacious treatment approaches and preventive measures. A deeper exploration is required to fully comprehend the scope of these trauma-epigenetic interactions in PTSD and chronic pain, which will ultimately offer a renewed sense of optimism for Veterans grappling with these conditions.

### Veteran parenting effects on development of adolescent chronic pain

To date there have been limited studies examining the relationship between Veteran PTSD and/or pain, parenting, and offspring outcomes. We must therefore draw insight regarding the influence of parenting style on childhood outcomes from other literature. Indeed, Veterans are parents, and as such do not experience PTSD/trauma in a vacuum. Research demonstrates that parental trauma influences child outcomes via neurobiological (epigenetics, stress hormones during pregnancy) and behavioural (parenting) pathways [126–128]. For example, animal studies clearly illustrate that toxic stress modifies early parental behaviours such as pup retrieval, licking, grooming, nesting, and feeding, all of which negatively influence offspring outcomes [129–132]. In addition, paternal PTSD has been linked to higher levels of family conflict [133] whereas paternal psychological distress has been linked to borderline problems as well as increased internalizing and externalizing symptoms in their adolescents, which was possibly mediated by their harsh and overprotective parenting styles [134]. Finally, parents who experienced childhood traumatic events are at increased risk of experiencing mental illness, negatively affecting their ability to engage in positive parenting behaviours [126–128]. Nevertheless, even in the face of trauma, there is resilience, both in parents and their children, and this is especially the case when comparing human versus animal studies [135, 136]. Despite the clear link found between parental trauma (ACEs) and offspring outcomes in rodents [135, 137], similar links have not been found in clinical samples. Research has shown that while the rates of parental ACEs are relatively high (particularly neglect) in parents of youth with chronic pain [138], this was *not* linked to worse chronic pain in children [126, 139]. Corroborating this, a recent study by Lund et al, demonstrated that although the offspring of Canadian Veterans experienced high rates of chronic pain, parental symptoms did not influence child symptoms, suggesting that their family dynamics possibly fostered resiliency within this cohort [140]. Nevertheless, the majority of research suggests that parental trauma (including ACEs) likely increases risk factors that tip the scales towards onset of chronic pain in their children.

Despite this however, one potential protective mechanism is social support. While the construct can be defined in various ways, social support encompasses tangible, emotional, and affectionate forms of assistance, in addition to fostering positive social interactions [141]. Research has shown that maternal HPA axis function in pregnant mothers was the mechanism by which maternal trauma influenced their infants’ later HPA axis reactivity. However, increases in social support buffered the association between mothers’ HPA axis function and infant cortisol reactivity [142]. Indeed, a recent review suggests that parents’ positive childhood experiences (which include social support) are protective against intergenerational risk of trauma in their children [143].

## Conclusion

Both Veterans and civilians living with PTSD are significantly more likely to report chronic pain, revealing a comorbid relationship between these two conditions [1, 3, 144]. The intergenerational paternal transmission of risk for chronic pain is a significant challenge that affects Veterans, highlighting our need to further understand the complex interaction between traumatic experiences resulting in PTSD and epigenetic processes [5]. The literature provides strong evidence that paternal PTSD leads to DNA methylation changes in genes related to glucocorticoids, specifically *NR3C1* and *FKBP5*, that correlate with offspring cortisol levels, glucocorticoid response, and symptom severity. Thus, epigenetic changes in fathers resulting from PTSD can be transmitted intergenerationally, leading to altered outcomes in offspring. For instance, we have previously demonstrated (Fig. 5) how such HPA axis dysregulation can lead to chronic pain. In addition, inherited epigenetic changes can produce phenotypes that are optimally programmed to thrive in the instigating environment. However, it is when the current environment does not match the expected (or programmed to) environment, that response to later stressors may be maladaptive, predisposing offspring to poor health outcomes, such as pain, anxiety, and psychological challenges [145–147].

Moving forward, future studies should aim to further characterise the epigenetic mechanisms that play a role in transmitting paternal epigenetic modifications from fathers to their children, with a focus on genes that are implicated in both PTSD and chronic pain. Additionally, research suggests that maltreatment and household dysfunction are also strong predictors of chronic pain [148]. These issues highlight the importance of further research into relationship between paternal experience, both prior to conception and during offspring development, and the long-term health outcomes, including the chronification of pain in their offspring.

As chronic pain is often difficult to treat, preventative strategies aimed at reducing PTSD and therefore epigenetic transmission of risk from Veterans to their children, should be explored. Current approaches to treating PTSD are still similar to those employed by mental health professionals for the management of “War Neuroses” during and after World War 2. More than 80 years ago, sodium pentothal was used in conjunction with exposure therapy to treat soldiers and Veterans suffering from “War Neuroses” [149]. Building upon these findings, and in an effort to improve outcomes and reduce negative side effects, 3,4-methylenedioxymethamphetamine (MDMA) assisted therapy is currently being used to treat soldiers and Veterans with PTSD, and has significantly reduced PTSD symptoms [150]. Additional contemporary therapeutic regiments with demonstrated efficacy include repeated ketamine infusions, which have effectively reduced PTSD symptom severity in a subset of soldiers with chronic PTSD [151]. However, given the negative risks associated with these psychedelic drugs, further research into pharmacological treatments for PTSD and chronic pain are needed. An interesting area of research that is emerging is the use of psychedelic compounds such as N, N-dimethyltryptamine (DMT) and ayahuasca/pharmahuasca (both contain DMT) for treating combat related PTSD. One study has shown that DMT and pharmahuasca reduced the expression of NF-κβ2 and inflammatory pathway cytokines [152], which is significant considering that PTSD and chronic pain conditions result in an upregulation of inflammatory pathways [115]. It has been suggested that DMT and ayahuasca promotes neurogenesis and increases synaptic plasticity by activating *SIGMAR1*, which interacts with HDAC1, 2, and 3 thereby modulating gene expression via chromatin compaction states [153]. For example, these epigenetic changes are involved in *SIGMAR1* activation (fear extinction), increased BDNF expression (fear extinction, memory consolidation) [153], and *FKBP5* upregulation involved in the stress response [154]. Exploring the ability of these pharmacologics to influence epigenetic processes may provide an avenue for the development of safer and more targeted therapeutics.

In summary, given that studies clearly demonstrate a relationship between paternal trauma and epigenetic modifications in offspring that ultimately modify risk or resiliency for later disease, there is a need to further understand this association. Although military personnel and Veterans experience significantly higher rates of trauma and PTSD, additional research can lead to evidence-based treatment strategies, that would allow us to break the intergenerational cycle risk and prevent chronic pain and other comorbid conditions in the next generation.
